# Dr. Hoys New Dental Lamp

**Published:** 1858-04

**Authors:** Jno. T. Toland


### A NEW SELF-ACTING BLOW-PIPE—DR. HOY'S DENTAL LAMP.

MR. TOLAND—DEAR SIR:—Agreeable to your request, I hand you below a description of my "Dental Lamp," which was gotten up by myself as a matter of necessity; but finding it so far superior to any thing I have met with in Dental Depots, I am induced to extend its advantages to the profession, and am now manufacturing some *for sale.*

The first one was made in Sacramento City for my own use, in 1853, and used with the utmost satisfaction and benefit, while remaining in California, during which time I was in the active practice of dentistry; and now after having thoroughly tested its practical utility, I offer it to the profession.

For usefulness and convenience, it has no equal as an alcohol soldering lamp, and it is perfectly adapted to all the various uses to which a lamp is put in a dental laboratory, combined in one convenient, portable form. It is made of copper, the body is 4¼ inches by 3. The main wick tube is 1⅛ inch, and the boiler wick tube ⅜ inch, which extends to near the bottom of the lamp. These tubes, are double, the outer one slides upon the other by means of a ratchet, which enables the workman to graduate the flames in the most perfect and easy manner imaginable, rendering it safe from the usual dangers of bursting, by having absolute control over it by this arrangement. The caps fit on these outside tubes closely, which prevents the waste of fluid and preserves the wick, which is seldom changed. The boiler is about 3 inches high by 2¼ wide at the base,—connical shape. The top is depressed perhaps ⅜ of an inch, and strengthened by the soldering on of extra plate, through which a hole ⅜ of an inch in diameter is drilled, and by this the boiler is filled and simply corked, and this held down by a steel spring constitutes the safety valve, and it is far better than any other arrangement in practice. The pipe passes from below upwards and backwards to the top. Its end is enlarged a little, and hollowed out into which it is fitted, the end of the *temper screw*, which is a thumb screw passing into the boiler, the head of wood, that the heat may not prevent it from being adjusted at will. The boiler with its pipe all moves forward and back, by a convenient arrangement on the top of the lamp, and also turns around with the slightest motion. By this means, the flame can be gentle or strong flaming (as in heating up a job), or the most beautiful point can be obtained by moving forward the boiler, and turning down the temper screw. With this lamp and a trailing wire, any kind of a job of soldering can be done with the greatest facility, and, sometimes without the use of the mouth pipe, or, with those who prefer it, heat up, turn round the boiler with the end of your blow-pipe, and finish the work with it.

It is only necessary, that the mechanical dentist should see and work with this lamp, to be made sensible of its great superiority over the Lawson Lamp, or any other now in use.

Note.—We have never seen one of the above described lamps, but if they come up to the representations, they will be a desirable addition to the Laboratory. We expect to have them for sale as soon as some of them are finished, and we presume they will be kept at all the Dental Depots. The price is not positively fixed, as the exact *cost* cannot yet be ascertained, but will be $12 or $15, more probably the latter.

JNO. T. TOLAND.

